# Prosthetic Joint Infection by Streptococcus lutetiensis (Bovis) With Noncancerous Polyps and Negative Blood Cultures: A Case Report

**DOI:** 10.7759/cureus.74264

**Published:** 2024-11-22

**Authors:** Ibrahim Shanti, Malik Samardali, Adamsegd I Gebremedhen

**Affiliations:** 1 Internal Medicine, Marshall University Joan C. Edwards School of Medicine, Huntington, USA

**Keywords:** ceftriaxone, large bowel diverticula, prosthetic knee joint infection, small bowel polyp, streptococcus bovis

## Abstract

Prosthetic joint infection (PJI), caused by Streptococcus bovis group (SBG), is uncommon and related to colorectal cancer. We present here a case of an 84-year-old male who had a past medical history of chronic obstructive pulmonary disease (COPD), congestive heart failure, pulmonary arterial hypertension, iron deficiency anemia, chronic kidney disease, diabetes mellitus, gout, hypertension, bilateral knee replacement with left knee pain and swelling. We initially suspected gout and treated him with prednisolone, but it did not relieve him. Joint aspiration revealed high WBC, predominantly neutrophils, and polymerase chain reaction (PCR)-identified Streptococcus lutetiensis. The patient underwent knee incision, drainage, poly exchange, and wound vacuum-assisted closure (VAC) placement. Sensitivity testing led us to treat him with ceftriaxone. Echocardiography revealed no endocarditis. A colonoscopy revealed multiple non-bleeding polyps and adenomas. After discharge, our patient completed six weeks of ceftriaxone via a peripherally inserted central catheter (PICC) line. At follow-up, our patient reported no complaints of fever and knee swelling.

## Introduction

S. bovis, often known as Streptococcus bovis, is a commensal habitant of the human digestive system. Up to 35% of fecal samples from human rectal swabs may contain it [1]. The relationship between S. bovis bacteremia and colorectal neoplasia has been well documented in the literature since the first notable case of the connection between an enterococcal infection and colorectal carcinoma, which was presented by McCoy et al. in 1951, and the case-control study by Klein et al. more than two decades later [2,3,4]. It has been estimated that S. bovis contributes roughly 6% of infectious endocarditis (IE), indicating that IE is a significant variable linked to the two occurrences [5,6]. With the development of more accurate identification methods, Streptococcus bovis group (SBG) nomenclature and disease associations have been modified to reveal a link between S. bovis biotype I (S. gallolyticus subsp. gallolyticus) and colorectal cancer [7,8]. However, little has been published on SBG septic arthritis, and even less has been recorded concerning SBG prosthetic joint infections (PJI) [9,10].

Streptococcus lutetiensis prosthetic joint infections are uncommon, but they can be dangerous if not recognized and treated right away. The case under discussion highlights an 84-year-old male with a history of gout and multiple comorbidities who arrived with left knee discomfort and swelling. Despite initial suspicions of gout, subsequent investigation revealed a Streptococcus lutetiensis-caused prosthetic joint infection. The current case is significant because it links Streptococcus lutetiensis to noncancerous gastrointestinal (GI) diseases like diverticulosis and polyps. The patient's history of tubulovillous adenomas in the colon, as well as multiple diverticula, suggests the presence of an underlying GI pathology that predisposes him to Streptococcus lutetiensis infection. It is critical for clinicians to examine these correlations when evaluating patients with prosthetic joints.

## Case presentation

An 84-year-old male patient presented with complaints of left knee pain and swelling that began approximately six days prior to the presentation. He suspected an acute gout flare due to his history of gout attacks and was initially treated with prednisone, which did not alleviate his pain. The patient denied experiencing fever, chills, rigor, or any other associated symptoms. He also denied recent trauma or injecting any material into his knee. The patient's past medical history included chronic obstructive pulmonary disease (COPD), congestive heart failure, pulmonary arterial hypertension, iron deficiency anemia, chronic kidney disease (CKD) stage 3A, diabetes mellitus, gout, and hypertension. He had a past surgical history of bilateral knee replacement 20 years ago and cardiac stenting. Socially, the patient denied smoking, drinking, or using illicit drugs. There was no previous family history of GI malignancy. Physical examination was significant for left knee swelling, redness, and warmth. Patient laboratory test results are given in Table 1.

**Table 1:** Laboratory result

Lab Test	Results	Reference Range
WBC count	14 x 10^3/µL	4.5-11 x 10^3/µL
Hemoglobin (Hb)	8 g/dL	13.8-17.2 g/dL (men) / 12.1-15.1 g/dL (women)
Potassium (k)	4.5 mmol/L	3.5-5.0 mmol/L
Sodium (Na)	137 mmol/L	135-145 mmol/L
Chloride (Cl)	104 mmol/L	98-107 mmol/L
Blood urea nitrogen	94 mg/dL	7-20 mg/dL
Creatinine	4.7 mg/dL	0.6-1.2 mg/dL
Calcium	8.8 mg/dL	8.5-10.2 mg/dL
Erythrocyte sedimentation rate (ESR)	116 mm/hr	0-20 mm/hr
C-reactive protein (CRP)	6.3 mg/L	<3.0 mg/L
Joint aspiration WBC	90,000/µL	<200/µL (normal synovial fluid)
Joint aspiration neutrophils	91%	<25% (normal synovial fluid)
Joint aspiration RBC	97%	Typically <2% in synovial fluid

X-rays of the left knee revealed total knee arthroplasty in place with no evidence of fracture or hardware failure. Orthopedics were consulted on admission due to concern for prosthetic joint infection, and the patient underwent joint aspiration, which revealed a white blood cell count of 90,000, with 97% RBCs and 91% neutrophil predominance. PCR testing was positive for Streptococcus, confirmed as Streptococcus lutetiensis on culture.

**Figure 1:**
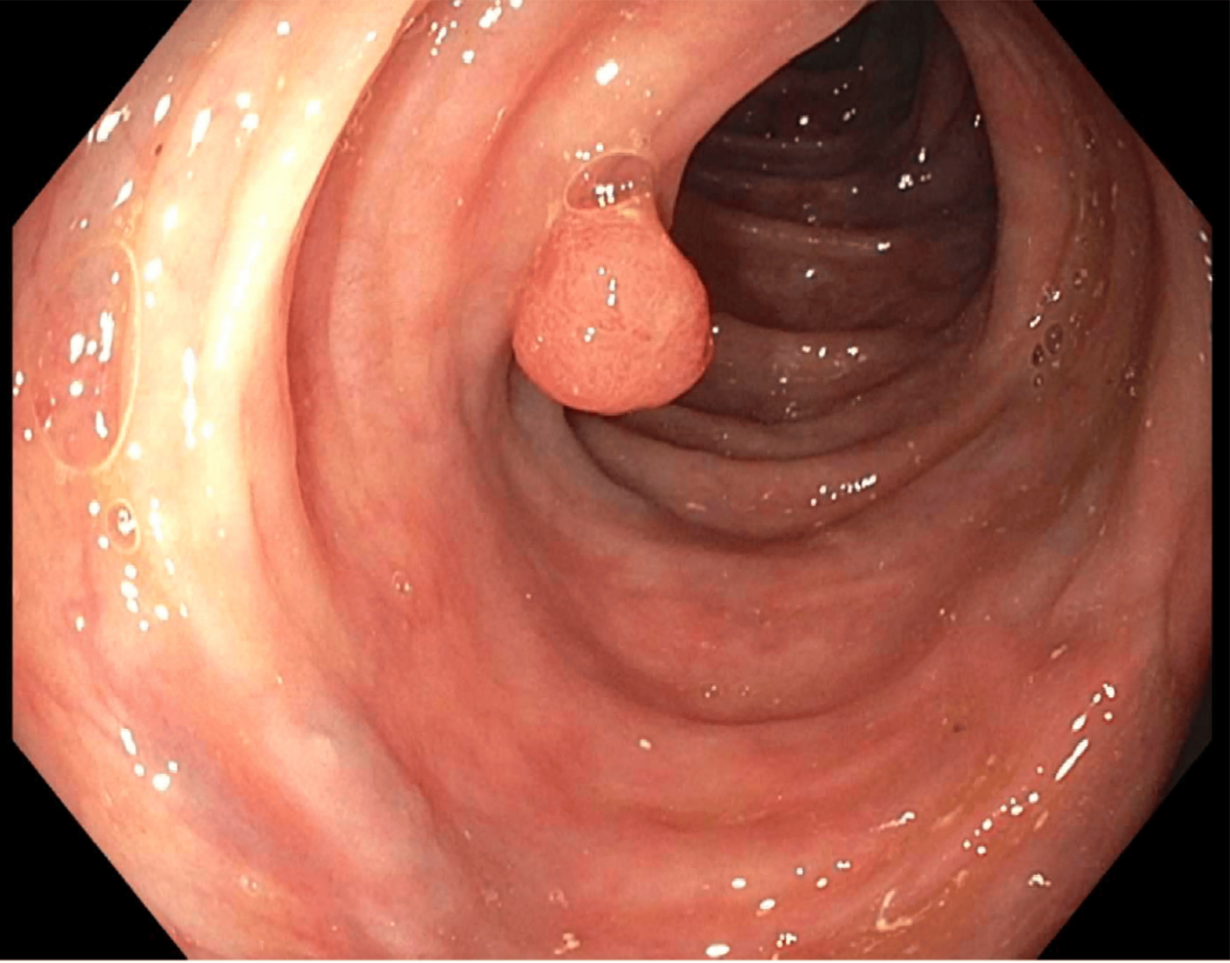
A colonoscopy showed a large polyp

**Figure 2:**
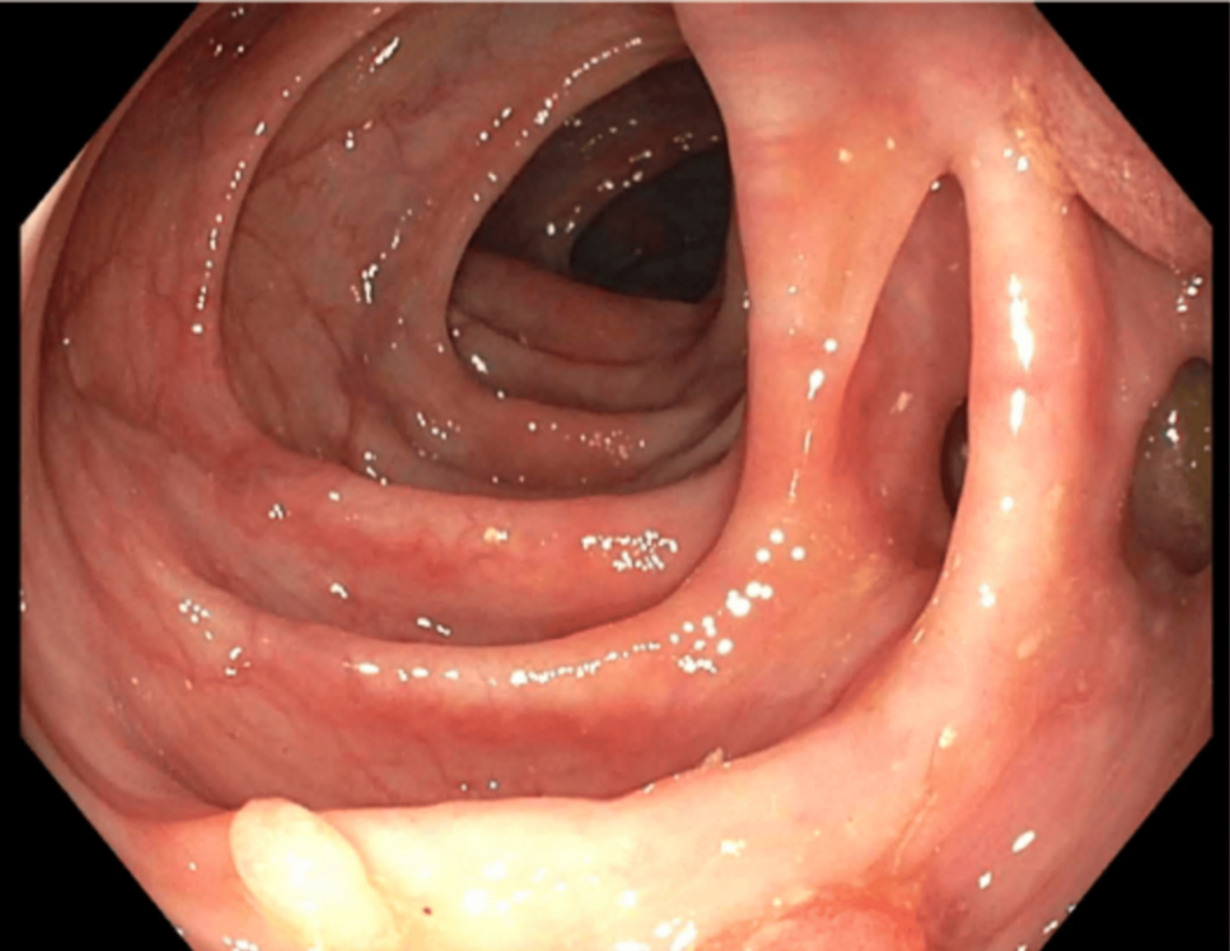
Colonoscopy showing multiple diverticulosis

The patient underwent incision and drainage with poly exchange and wound vacuum-assisted closure (VAC) placement. Sensitivity testing showed the Streptococcus lutetiensis strain to be sensitive to ceftriaxone, so the patient was started on ceftriaxone. Blood cultures from two bottles were negative. Due to concerns of malignancy and endocarditis, the patient underwent an echocardiogram, which showed no vegetation. The patient had a colonoscopy three years ago, which showed diverticulosis in the descending colon with no other polyps or ulcers. The patient was advised to repeat the colonoscopy given the risk of malignancy. A colonoscopy was done, which revealed multiple non-bleeding polyps in the cecum and multiple diverticulosis all over the colon, with two large polyps (14mm and 16mm) in rectosigmoid biopsy showing tubulovillous adenomas. No dysplasia and no malignancy were noted (Figures 1, 2). According to Paris classification and NICE type 2 on narrow band imaging, 0-1ps polyp was noted.

The patient was prescribed ceftriaxone by the infectious disease team for arthritis treatment; a peripherally inserted central catheter (PICC) line was inserted, and the patient was sent home with ceftriaxone for six weeks. After completing the antibiotic course, the patient reported no fever, chills, or swelling in the knee upon further evaluation.

## Discussion

For a long time, it has been known that S. bovis-group bacteremia and premalignant adenomatous polyps or underlying gastrointestinal cancers are clinically related [2,3]. There is inadequate understanding of this among medical professionals, despite the fact that this association has significant therapeutic consequences for patients and physicians imposed with continuing care of these patients both during serious illnesses and afterward. An unacceptable low proportion of individuals (only 52%) underwent gastroenteroscopic examination upon hospital discharge [11]. But our patient underwent colonoscopy, which revealed multiple non-bleeding polyps in the cecum and multiple diverticulosis all over the colon, with two large polyps (14mm and 16mm polyps) in rectosigmoid biopsy showing tubulovillous adenomas and no malignancy. Tumors of benign neoplastic epithelium with varying degrees of malignancy potential are known as adenomatous polyps. It is widely acknowledged that the adenoma-carcinoma sequence is responsible for the genesis of over 95% of colorectal malignancies [12].

Few studies have been conducted concerning S. bovis PJI, despite knowing that SBG, more especially S. bovis biotype I (S. gallolyticus) infections, such as bacteremia and/or infective endocarditis, have been linked to colorectal cancer [11]. Right now, colorectal cancer ranks as the fourth most frequent cancer in the US. Although colorectal cancer patients only have a 65% overall survival rate, research has demonstrated that early polyp removal, detection, and effective therapy all improve survival [13,14]. It has been demonstrated that colorectal cancer modifies the natural gut microbiota, which encourages the growth of Streptococcus gallolyticus [15]. According to certain theories, this bacteria secretes inflammatory proteins that promote long-term inflammation, prevent apoptosis, and boost angiogenesis, all of which might result in the development of tumors [16]. As a result, SBG PJI might be useful in the early detection of colorectal cancer.

One specific way that S. lutetiensis can spread to a joint is through an abnormality in the gastrointestinal mucosal tract that makes the bacteria more likely to translocate into the bloodstream. From there, the bacteria can infiltrate and form a biofilm on a prosthetic joint, which is how S. bovis bacteremia happens. In the literature, there are six previous case reports of SBG PJI, in addition to a case series involving eight patients [17]. Nine PJI in eight patients were found, with a mean prosthesis age of eight years at diagnosis (with a range of four weeks to 17 years). The range of 0.3 weeks to 175 weeks represented the median time between the onset of symptoms and the start of treatment. Of them, 8/9 had their PJI completely resolved with acuity-based care. Debridement, antibiotics, and implant retention (DAIR) were used to treat two cases of acute PJI, and two stages of revision arthroplasty were used to treat seven cases of chronic PJI. After first receiving treatment, one patient with persistent PJI experienced recurrent infections. Every patient received IV antibiotics post-op. Ceftriaxone was given to seven out of eight patients. Three individuals were prescribed oral antibiotics for life. Seven or eight patients had colonoscopies. Following PJI diagnosis, polyps were discovered in 5/7 patients, including two dysplastic and one carcinomatous polyp. Diverticulosis, long-term anticoagulation use, and a history of tooth extraction prior to symptom onset were the two notable gastrointestinal (GI) mucosal abnormalities observed in the two individuals without polyps [17]. Of the six cases that were documented, four had concurrent colorectal cancer, and the other two had either endocarditis or premalignant polyps [9,10,18,19,20]. Our patient had a knee incision, wound VAC installation, drainage, and poly exchange. The infectious disease team administered ceftriaxone for arthritis treatment, placed a PICC line, and discharged our patient on ceftriaxone for six weeks. Following the antibiotic treatment, our patient experienced no fever, chills, or knee swelling upon additional examination.

We hope that our case will be a valuable addition to the existing literature.

## Conclusions

The most common cause of SBG PJI is hematologic seeding. Proper referrals to gastroenterologists should be made in order to assess the possibility of indolent colorectal cancer. In the setting of a PJI, it is unlikely that cancer treatment will ensue. While colonoscopy should be emphasized, the timing of it need not be before surgical management of PJI. Routine colonoscopy is often deferred to six months after an arthroplasty as there is concern for translocation of bacteria with insufflation during colonoscopy that could seed the arthroplasty. It is recommended that people with SBG PJI pursue colonoscopy. A six-week regimen of ceftriaxone combined with surgical intervention determined by the severity of the infection appears to be adequate to control it. In short, this case emphasizes how crucial it is to identify the association between benign gastrointestinal conditions, such as diverticulosis and polyps, and Streptococcus lutetiensis which has been noted in patients presenting with infections in prosthetic joints. Our patient responded well to a six-week course of ceftriaxone treatment with no recurrence of knee swelling. Gaining insight into these relationships can assist direct clinical care and enhance patient outcomes.
